# An ISE-based On-Site Soil Nitrate Nitrogen Detection System

**DOI:** 10.3390/s19214669

**Published:** 2019-10-28

**Authors:** Yanhua Li, Qingliang Yang, Ming Chen, Maohua Wang, Miao Zhang

**Affiliations:** 1 Key Laboratory on Modern Precision Agriculture System Integration Research of Ministry of Education, China Agricultural University, Beijing 100083, China; 15001228421@163.com (Y.L.); qingliangyang@cau.edu.cn (Q.Y.); mercury@cau.edu.cn (M.C.); wangmh@cau.edu.cn (M.W.); 2 Key Lab of Agricultural Information Acquisition Technology of Ministry of Agriculture and Rural Affairs, China Agricultural University, Beijing 100083, China

**Keywords:** on-site detection, ion-selective electrode (ISE), soil nitrate nitrogen (NO_3_^−^-N), soil moisture, sensor fusion

## Abstract

Soil nitrate–nitrogen (NO_3_^−^-N) is one of the primary factors used to control nitrogen topdressing application during the crop growth period. The ion-selective electrode (ISE) is a promising method for rapid lower-cost in-field detection. Due to the simplification of sample preparation, the accuracy and stability of ISE-based in-field detection is doubted. In this paper, a self-designed prototype system for on-site soil NO_3_^−^-N detection was developed. The procedure of spinning centrifugation was used to avoid interference from soil slurry suspension. A modified Nernstian prediction model was quantitatively characterized with outputs from both the ISE and the soil moisture sensor. The measurement accuracy of the sensor fusion model was comparable with the laboratory ISE detections with standard sample pretreatment. Compared with the standard spectrometric method, the average absolute error (AE) and root-mean-square error (RMSE) were found to be less than 4.7 and 6.1 mg/L, respectively. The on-site soil testing efficiency was 4–5 min/sample, which reduced the operation time by 60% compared with manual sample preparation. The on-site soil NO_3_^−^-N status was dynamically monitored for 42 consecutive days. The declining peak of NO_3_^−^-N was observed. In all, the designed ISE-based detection system demonstrated a promising capability for the dynamic on-site monitoring of soil macronutrients.

## 1. Introduction

The ion-selective electrode (ISE) transfers the ionic activity (or concentration) of the target ion dissolved in testing solutions into electromotive force (EMF). Theoretically, the measured EMF is related to the logarithm of the ionic activity according to the Nernst equation. Because of the importance of fertilizer in agricultural production, ISEs have been used in soil nitrate–nitrogen (NO_3_^−^-N) analysis for more than half a century [1]. A prototype ISE based on an in-field nitrate monitoring system was first developed in 1994 and has been successively improved by Canadian researchers [2,3,4]. Soil samples were collected at a depth of 0–15 cm with an autosampler. GPS information was recorded at the same time. Programmable processes of soil bulk crushing and plant residue removing were designed. NO_3_^−^-N extraction was obtained by mixing the collected soil with de-ionized distilled water (DDW). The influence of soil texture was considered in sensor calibration. The fifth generation of the modified system demonstrated a satisfactory correlation with the standard method. An R^2^ of 0.92 was found in testing of 13 sets of samples. The problem of random ISE signal disturbance caused by soil slurry was claimed. 

In 2001, a portable ISE detection kit was developed for direct in-field measurement of soil chemical properties, including pH, mineral Na^+^, mineral K^+^, and NO_3_^−^-N [5]. More than 500 soil samples were collected. However, the NO_3_^−^-N testing results demonstrated obvious variations from the standard spectrometric method. At the same time, researchers from the University of Missouri compared extractants for ISE-based soil macronutrient detection. Kelowna solution was chosen for the extraction of soil available K^+^, PO_4_^3−^, and NO_3_^−^-N. Extracted soil solution was manually obtained using the recommended soil testing protocol. Feasibility was evaluated with 37 samples. ISE based laboratory soil NO_3_^−^-N detection demonstrated good accuracy with standard deviations ranging from 8.04 to 19.7 mg/L [6,7]. Multiple studies were conducted on ISEs foron-the-go soil macronutrient monitoring by Adamchuk et al. For the purpose of achieving on-the-go soil testing, the “Direct Soil Measurement” (DSM) system was designed and then validated, updated, and commercially transformed in 2005. The ISEs of NO_3_^−^, K^+^, and pH were integrated to form the sensing unit. De-ionized(DI) water was applied for the cleaning of the ISE sensing array. Sensing results were directly collected without pretreatment operations of stirring and filtration. Compared with laboratory detection, the DSM results of NO_3_^−^, K^+^, pH were reported with coefficients of determination (R^2^) of 0.41–0.51, 0.61–0.62, and around 0.9, respectively [8,9]. Insufficient sample extraction was considered to be a possible reason for the unsatisfactory accuracy level. Sethuramasamyraja et al. improved the soil pretreatment process of the system by integrating a mechanical agitation operation into the sample extractant process. The “Integrated Agitated Soil Measurement” (ASM) results of the soil pH were comparable to laboratory testing with an R^2^ value of 0.99. However, the predicted NO_3_^−^ value still demonstrated great deviation from standard spectrometric results with an R^2^ value of 0.48 [10]. On the basis of the ASM system, the latest “On-the-Spot Analyzer” (OSA) system was developed for the simultaneously measurement of soil properties at a predefined soil depth. ISEs were brought into direct contact with the conditioned soil slurry, after the testing stand was moved to the experimental field and the topsoil was removed. Once sensors readings were retrieved, the analyzer was removed to another testing spot. Forty-five sets of surface topsoil samples with NO_3_^−^-N concentrations ranging from 0 to 30 mg/kg were measured on the spot. The correlation coefficient R^2^ was increased to 0.87 [11]. The improved detection accuracy with the OSA system demonstrated promising potential for the achievement of automated measurements. 

As far as we are concerned, most of the in-field soil testing discussed above involves reduced soil pretreatment operations due to the system’s simplicity and efficiency. The testing error, produced by “soil particle suspension disturbance”, reached a magnitude of 26.6 mg/kg with an average relative error of 50% according to our preliminary laboratory validation of ISE-based NO_3_^−^-N detection with 15 soil samples [12]. Besides, soil slurry would contaminate the membrane of ISE. The response slope of NO_3_^−^ ISE was determined to be 44.4 and 25.4 mV/decade after continuous testing for 4 and 12 h, respectively [13]. Thus, it was necessary to obtain a transparent soil extract to enhance the accuracy and lifetime of the ISE. Pan et al. [14] tried to separate the clear soil NO_3_^−^-N extractant from sample slurry through the short-time process of spinning centrifugation. Seven soil samples were used for the optimization of the centrifugation operation. Clear soil extractant was obtained by spinning for 30 s at the centrifugation speed of 1000 rpm. Compared with the direct soil slurry detection, the NO_3_^−^-N detection relative error decreased from 64% to 5%. Yanhua et al. [15] attempted to evaluate the effects of uncalibrated soil moisture on NO_3_^−^-N with six samples at the laboratory. The moisture of the tested samples was pre-manipulated to 2%–25%. The ISE based NO_3_^−^ ISE results were uniformly smaller than the standard spectrometric results when the influence of soil moisture was neglected. A soil moisture percentage of 25% produced a maximum absolute error of 30 mg/kg. An error of no less than 5.0 mg/kg occurred even when the soil moisture was 5%. 

For the purpose of improving the accuracy of on-site soil NO_3_^−^-N detection, a self-designed prototype system was designed by making use of the sensor fusion method. Both the NO_3_^−^ ISE and soil moisture sensor were employed as the sensing unit. The specific objectives were, first, to integrate necessary soil pretreatment steps, e.g., sample weighting and extractant spinning centrifugation into an on-site testing bench. Second, we investigated a modified Nernst model for the prediction of soil NO_3_^−^-N with the real-time data provided by the ISE and the moisture sensor. Finally, we evaluated the feasibility of the system.

## 2. Materials and Methods

### 2.1. Reagents and Apparatus

A soil moisture sensor (ECH2O-5TE, Decagon, WA, USA) produced volumetric moisture readings that were used to determine the soil’s net weight. The sensor was claimed to have a detection precision of ±3% m^3^/m^3^. Reagents used were all Analytical grade. The testing solution was prepared with Deionized Water (Di-water). Standard soil chemical properties were provided by the soil testing center of the China Agricultural University with commercial analytical instruments. Detection was carried out according to the guidance of soil testing and fertilizer recommendations [16]. Soil moisture was oven dried at the temperature of 65 ℃ for 8 h (SG-GDJ50, SIOM, Shanghai, China). Soil NO_3_^−^-N was detected with a UV-VIS spectrometer (UV2450, SHIMAZU, Kyoto, Japan) at 210 nm. H_2_SO_4_ (70%) was applied to the soil extractant for acidification. The Total-N (TN) soil concentration was determined with Kjeldahl determination (KJELTEC 8400, FOSS, Hillerød, Denmark). Soil available phosphate (AP) was detected based on Molybdenum Blue Colorimetry at 660 nm (UV2450, SHIMAZU, Kyoto, Japan). The Organic Carbon (OC) concentration was measured based on dry combustion at 550 ℃ for 24 h (SG-SJ1700, SIOM, Shanghai, China). Flame photometry (420, Cole-Parmer, IL, USA) was used to measure the Available potassium (AK) content of the soil. Commercial nitrate ISE (No.9707BNWP, Thermo Scientific Orion, MA, USA) with a detection limit of 1.4 mg/L was also employed in this study.

The analytical grade chemicals used for the calibrations of ISE and the detection of standard soil macronutrients were purchased from Sinopharm Chemical Reagent Beijing Co. Ltd.

### 2.2. Sensor Fusion Model

The detected NO_3_^−^-N content would be greatly underestimated if soil moisture interference was not involved in the compensation of the sample net weight. In this study, volumetric soil moisture information was obtained during the on-site soil sampling. The volumetric moisture was converted into the gravimetric moisture for the correction of the sample’s net (dry) weight. The detailed procedure was discussed in a previously published paper [15]. A sensor fusion model was designed for the NO_3_^−^-N prediction, as illustrated in Equations (1)–(3). Compared to the conventional Nernst model, the ratio of extractant to soil weight of the sensor fusion model achieved real-time correction instead of using a constant value, as used in most of the previous studies.

(1) $$\omega =\frac{\rho_{w}\times (\theta -\theta_{0})}{\rho_{s}}=\frac{1}{\rho_{s}}\times (\theta -\theta_{0})$$

(2) $$N=\omega +\frac{\omega m+m}{M}$$

(3) $$C_{i}=1000N\cdot A_{r}10^{\frac{E-E_{0}}{S}}$$

where *ρ_s_* represents the pre-determined bulk density of dry soil (1.19 g/cm^3^); *ρ_w_* represents the density of deionized water (1.0 g/mL); *θ*_0_ represents the pre-determined volumetric moisture ratio (–1.51%); *θ* represents the soil volumetric moisture (%); *ω* represents the soil mass moisture (%); *M* represents the weight of the raw soil sample (g); *m* represents the volume of soil extractant (mL); *N* represents the ratio of extractant to the net weight of soil (mL/g); *Ar* represents the relative atomic mass, which, for nitrogen, is 14; $C_{i}$ represents the concentration of nitrate in the tested sample (m/V, mg/L); *E* represents the EMF value produced by ISE (mV); *E*_0_ represents the intercept potential of the Nernstian model of the tested ISE (mV); and *S* represents the response slope of the Nernstian model of the tested ISE (mV/decade), where decade means 10 times the change in the target concentration.

### 2.3. System Design

**Figure 1:**
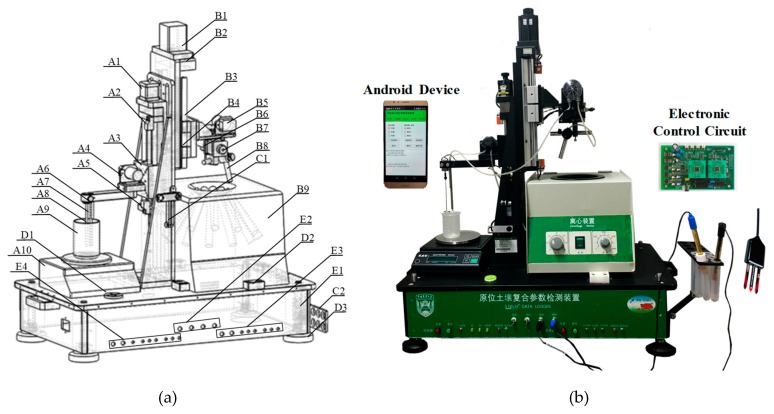

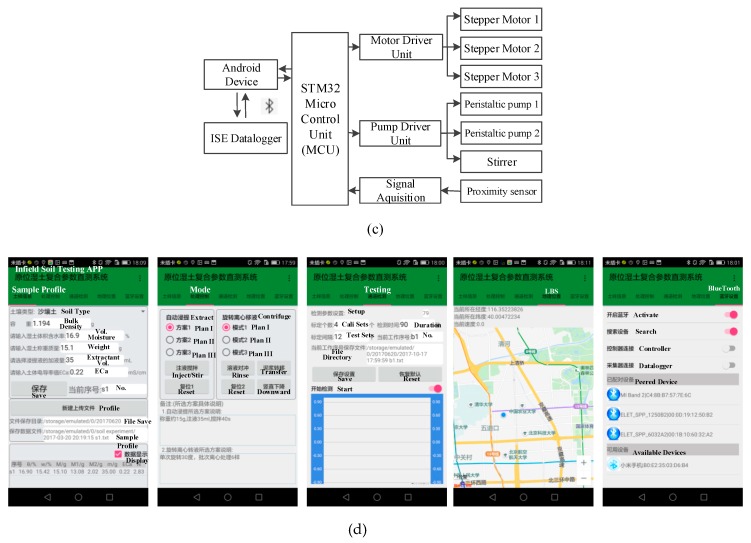
Diagram of the on-site detection bench: (**a**) System Design A1, Stepper motor 1 A2. Proximity sensor 1 A3, Vertical slide table 1 A4, Peristaltic pump A5, Proximity sensor 2 A6, Injecting tube A7, Outlet tube A8, Blender A9, Soil sample container A10, Electronic weight scale B1, Stepper motor 2 B2, Proximity sensor 3 B3, Vertical slide table 2 B4, Proximity sensor 4 B5, Peristaltic pump2 B6, Rotary table B7, Stepping motor 3 B8, Pipe hanger B9,Centrifuge C1, Electrode hanger 1 C2, Electrode hanger 2 D1, Horizontal Lever meter D2, Positioner D3, Leveling screw E1, Circuit controller E2, ISE connector E3, Control switches and indicator lights E4, Control switches and indicator lights; (**b**) Physical picture of the hardware; (**c**) Diagram of the Electric Control Circuit Design; (**d**) Android App for Smartphones.

The on-site soil NO_3_^−^-N detection bench consisted of five major units, including the extractant preparing unit (A), extractant clarification unit (B), electrode holder unit (C), leveling unit (D), and electronic control circuit unit (E), as illustrated in Figure 1a,b. Centrifuge (B9) was employed to achieve separation of the clarified extractant from the soil slurry. The centrifuge process was conducted at a speed of 1000 rpm for 3 min. The manually collected soil sample was weighed with electronic scales with a precision of 0.1 g (A10). Stepper motors of A1 and B1 were employed to achieve vertical movements of two mechanical arms for extractant injection and transportation. The proximity sensors of A5 and B4 were used to define the working scale of the vertical slide table (A3/B3). The precision of vertical movement was measured to be 0.05 cm. Rotary table B7 was driven by step motor B7. Centrifuge B9 had 12 container positions, so B7 would rotate by 30 each time with a control precision of 0.5°. Transportation of DDW and the sample extract was achieved by peristaltic pumps A4/B5 through tubes of A6/A7. The stirring operation was performed with Blender A8. ISE testing was conducted by hanging the sensor on C2. To keep the balance of A10 and B9, the bench employed leveling meter D2, positioner D3, and screw adjuster E1. 

The detection bench was manipulated in a programmable way by the self-designed electronic control circuit unit, as shown in Figure 1c. The STM 32 Microchip Controller Unit (MCU) was applied as the main processor. The underlying hardware of step motors 1–3 and peristaltic pumps 1–3 were motivated with the drive unit according to the pre-designated flowchart. A proximal sensing signal was sent to the MCU when the mechanical arms were close to the vertical limitation of 10 cm. A Bluetooth connection was formed among the control circuit, ISE datalogger, and Android terminal devices, e.g., smartphones. Sensor readings and user commands were communicated. A schematic diagram of the circuit is illustrated in Figure 1c.

The rural smartphone popularity was reported to be 32% in China [17]. Considering the interface resource, flexible communication mode, convenient data storage, and upload capability, application software running on Android terminal devices was also developed in this study. The interface of the smartphone App is shown in Figure 1d. Predetermined soil sample profile information, including soil texture, bulk density, sample weight, DDW volume, and electroconductivity, should be input, saved, and downloaded to the control circuit. The parameters of the sample pretreatment operation, e.g., stirring time, rinsing method, and motor speed, are chosen according to the testing mode. Testing setups were employed with the calibration solution number, testing duration, sample number, file save option, and real-time display. A Location-Based Service (LBS) was embedded to provide the sample’s geographic position. The Bluetooth setup was operated on the App. 

### 2.4. Field Test Design

**Figure 2:**
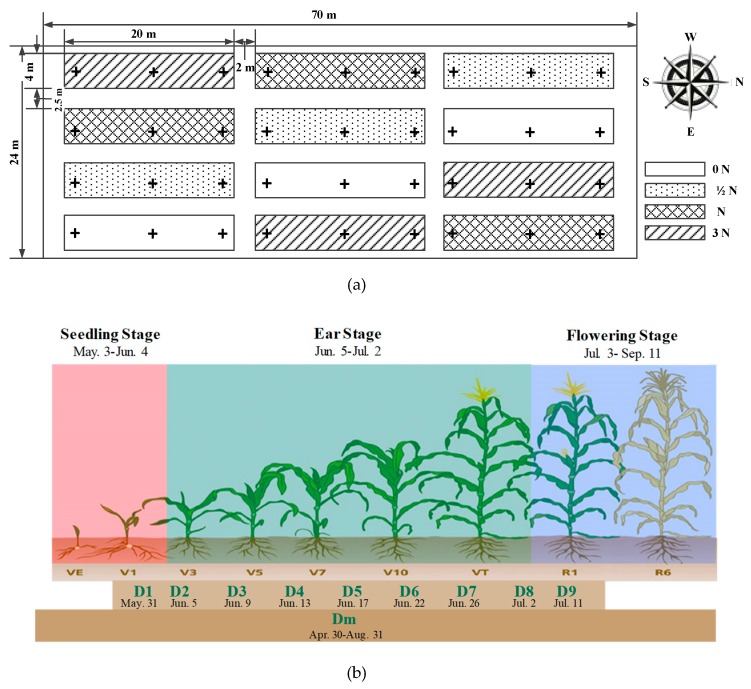
Soil Sampling Information: (**a**) Sampling space position inside the field (**b**) Sampling time.

Fresh soil samples were manually collected at a depth of 0–25 cm from a demonstration summer corn planting farm (70 L × 24 W m^2^) from April 30 to Aug 31, 2016 (40°8′37″ N, 116°11′31″ E). Soil sampling information is shown in Figure 2. The cornfield was divided into 12 fertility zones with a varied N application rate from 0 to 3 N, where 1 N equals the application of 375 kg/ha of compound fertilizer (Total content ≥ 40%, N:P_2_O_5_:K_2_O, 28%:6%:6%, Shidanli Co. Ltd., Shandong, China) and 75 kg/ha of urea; ½ N represents half of the 1 N rate; 0 N means no fertility; and 3 N means triple the rate. A total of 11 groups of soil samples were collected. Raw soil samples, detected in the field by the self-designed bench without moisture compensation, were recorded as ISE_raw_. ISE results, provided by the self-designed detection bench by the sensor fusion model, were recorded as ISE_OS_. Laboratory ISE soil testing results were labeled ISE_LT_, in which soil samples was treated with conventional soil pretreatments. Soil samples measured by the standard UV-VIS spectrometer were provided by the soil testing center of China’s Agricultural University. The nitrate–nitrogen content was recorded to be Stand_Spec_. 

**Figure 3:**
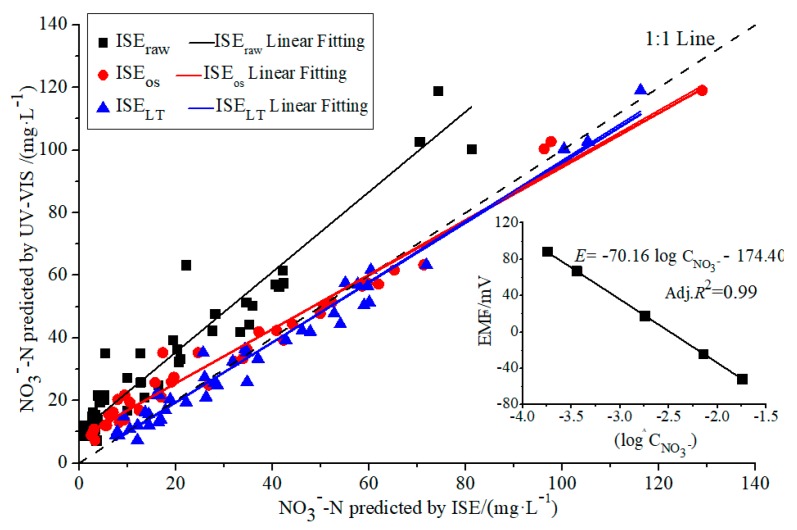
Comparison of soil NO_3_^−^-N predicted with ISE_raw_, ISE_OS_, and ISE.

Forty-two sets of raw samples, labeled as D_m_, with broader time variance, were randomly sampled in the field from April 30 to August 31. The D_m_ testing group was used to evaluate the performance of the designed sensor fusion model. Differences among Stand_Spec_, ISE_raw_, ISE_OS_, and ISE_LT_ were compared. The evaluation results are illustrated as Figure 3.

As demonstrated in Figure 2a, three sampling positions were marked with the plus cross icon in each of the 12 zones. One representative soil sample per zone was obtained by thoroughly mixing these three cores. A total number of 108 sets of fresh soil samples were collected for 42 days, which covered the summer corn growth stages from trifoliate to silking. The first 12 samples were collected on May 30, which were labeled as group D1. Then, the 7 continuous groups of samples, marked D2–D8, were obtained from June 5 until July 2, commonly at intervals of 3 days. The last group of soil samples (D9) was collected on July 11. Soil samples were applied to validate the feasibility of the on-site NO_3_^−^-N testing system. 

The soil properties provided by the standard testing center are summarized in Table 1. 

## 3. Results and Discussion

### 3.1. Validation of the Sensor Fusion Model

The sensor fusion compensation model, described in Equations (1)–(3), was evaluated with 42 soil samples, as demonstrated in Figure 3. The soil testing results of ISE_raw_ were, on average, 46.8% smaller than Stand_Spec_. The maximum deviation was calculated as 44.8 mg/L. ISE_OS_ and ISE_LT_ demonstrated a good correlation with the standard spectrometric results. Absolute error values of 0.2–17.2 and 0–9.8 mg/L were obtained, respectively. The measurement accuracy of ISE_OS_ was increased by more than 50% compared with that of ISE_raw_. The soil moisture compensation model eliminated the testing error. 

### 3.2. Evaluation of the On-Site Soil NO_3_^−^-N Detection

Soil NO_3_^−^-N detection results were compared among three different methods—standard spectrometric results, laboratory ISE testing, and on-site ISE based monitoring—as shown in Table 2. The testing efficiency was also evaluated. The time duration and the labor force consumed for dealing with a dozen soil samples were compared among UV-VIS, ISE_OS_, and ISE_LT_. The results are summarized in Table 3.

As illustrated in Table 2, the linear regression fitting results of ISE_OS_, ISE_LT,_ and UV-VIS were *y_UV-VIS_* = 1.02*ISE_OS_* − 0.57, *y_UV-VIS_* = 0.98*ISE_LT_* − 0.71. Both linear fitting curves were close to the 1:1 line. The ISE detection accuracy demonstrated a slight variation with the change in soil NO_3_^−^-N content. The accuracy was derived as ±30%, ±16% and 5% (Full Scale, FS) at the NO_3_^−^-N content ranges of 0–30, 31–90, and 91–200 mg/L, respectively. The maximum error (with the possibility of ±90%) was less than 10 mg/L. The intersection was close to 1. Adj. R^2^ values were both 0.98. The ISE results demonstrated close consistency with UV-VIS. The absolute error values among ISE_OS_, ISE_LT,_ and UV-VIS were calculated to be 0.1–19.9 and 0.0–18.4 mg/L with average values of 4.7 and 4.0 mg·L^−1^, respectively. The RMSEs were found to be 6.1 and 5.5 mg/L. No significant difference was found between the results of ISE_OS_ and ISE_LT_. 

The ISE_OS_ demonstrated obvious advantages in terms of the testing efficiency and labor force intensity, as shown in Table 3. Compared with the conventional soil pretreatment protocols conducted before UV-VIS and ISE_LT_, the self-designed on-site detection bench was decreased by 45 mins. The total time consumption was reduced to 40% of the duration of the conventional spectrometry method.

Integrated with the multi-sensor, centrifuge filtration, and programmable fluidic control, the self-designed on-site soil NO_3_^−^-N detection bench produced a reliable result with an efficient operation, which demonstrated a promising perspective for the infield monitoring applications.

### 3.3. NO_3_^−^-N Variation Monitoring

**Figure 4:**
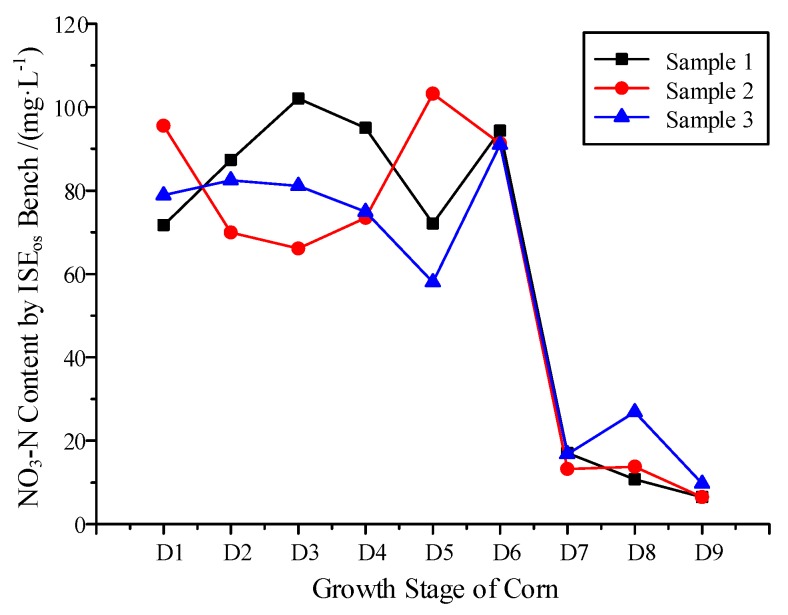
Monitoring of NO_3_^−^-N Variation by the On-site detection Bench.

Based on the workbench, the on-site NO_3_^−^-N variation was monitored from the trifoliate stage to the silking stage of summer corn. Samples collected from three 1N zones were selected to demonstrate the NO_3_^−^-N content change with corn growth, as shown in Figure 4. The NO_3_^−^-N content was at a level of around 70–100 mg/L at the beginning of D1. NO_3_^−^-N demonstrated great variation in characteristics with time and at different sample sites. However, an obvious NO_3_^−^-N decrease occurred uniformly at an amplitude of 80 mg/L across all three testing sites from D6 to D7. According to the definition of corn growth, D6 was the V_T_ period and D7 was in the R_1_ period, as shown in Figure 2b. The monitoring results perfectly fit the nitrogen growth law of corn. After that growth stage, no clear nitrogen absorption was verified. The NO_3_^−^-N content stayed at the level of 13.2–17.0 mg/L. 

## 4. Conclusions

In this paper, a self-designed prototype system for on-site soil NO_3_^−^-N detection based on ISE was designed and tested. Sensor fusion of ISE and a moisture sensor effectively eliminated 50% of the testing error. The performance of the on-site soil NO_3_^−^-N system demonstrated good consistency with the UV-VIS testing and laboratory ISE testing methods. Compared with the UV-VIS method, the average absolute error was determined to be 4.7 mg·L^−1^. The RMSE was found to be 6.1 mg/L. In addition, the detection duration decreased to 40% of that of the spectrometric method. 

## Figures and Tables

**Table 1:** Soil sample information.

	No.	Mass Moisture	Nitrate Nitrogen (mg·L^−1^)	Total -N ^1^ (g·kg^−1^)	Available-P ^1^ (mg·L^−1^)	Organic Matter ^1^ (g·kg^−1^)	Available-K ^1^ (mg·L^−1^)
D_m_	42	2.5%–30.2%	11.2–87.7	0.3–10.5	9.8–32.5	3.2–9.0	8.3–121.3
D1	12	12.5%–16.3%	33.1–159.8	0.3–9.9	2.4–43.3	1.3–11.2	10.3–98.8
D2	12	13.3%–16.9%	31.6–345.0	-	-	-	-
D3	12	13.2%–17.6%	27.5–272.0	-	-	-	-
D4	12	11.4%–15.4%	16.2–189.7	-	-	-	-
D5	12	10.6%–13.7%	19.3–260.5	-	-	-	-
D6	12	9.2%–15.1%	19.3–256.9	-	-	-	-
D7	12	23.8%–26.4%	12.9–72.3	-	-	-	-
D8	12	14.3%–17.3%	9.5–32.6	-	-	-	-
D9	12	14.8%–18.1%	5.2–16.7	-	-	-	-

^1^ Soil Total-N, Available-P, Organic Matter, and Available-K were tested in two groups of soil samples. D_m_ was 42 soil samples evaluated using the sensor fusion model. D1 was 12 samples used for the evaluation of the on-site bench. Detection was not conducted in D2–D9, because these soil properties were considered to be stable during the same corn growth season.

**Table 2:** Statistical analysis of the linear regression fitting results.

	Detection Range (mg·L^−1^)	Linear Fitting Model	*Adj. R* ^2^	*F-Value*	*P-Value*	*Sig.*	AE (mg·L^−1^)	MRE (%)	RMSE (mg·L^−1^)
ISE_OS_	5.0–156.3	y = 1.02x − 0.57	0.98	6055.8	0.0	*	0.1–19.9	13.9	6.1
ISE_LT_	5.9–150.5	Y = 0.98x − 0.71	0.98	5488.9	0.0	*	0.0–18.4	13.7	5.5

* represents that the linear fitting model is significant.

**Table 3:** Comparison of the testing duration and labor force among Stand_Spec_, ISE_OS_, and ISE_LT._

Measurement ^1^		Stand_Spec_		ISE_LT_		ISE_OS_	
Testing Duration (min) ^2^	OPERATIONS	Quantitative Weighing	12	Quantitative Weighing	12	Sample Weighing	2
		Extractant adding	12	Extractant adding	12	Extractant Injection	16
		Shaking	20	Shaking	20		
		Stabilization	20	Stabilization	20	Centrifuge Filtration	3
		Filtration	4				
		Titration	24	Filtration	4		
		Detection	15	Detection	24	Detection	24
	Total		107		92		45
Labor force Intensity		Intensive physical work. Participation in the overall process		Intensive physical work. Participation in the overall process		Light physical work. Participation in sample pickup and weighting.	

^1^ Soil samples detected by Stand_Spec_ and ISE_LT_ should be pretreated according to the soil testing recommendations. The shaking time required is 20 min. The optimal stabilization time is 20 min.; Soil samples detected by ISE_OS_ did not undergo quantitative weighting. Fresh soil samples were first weighed after moisture measurement. A peristaltic pump was used for extractant injection. The extractant injection rate was 36 s/sample. The stirring process was used for 40 s/sample. The centrifuge filtration rate was 40 s/12 samples. A stable ISE reading was obtained when the variation of EMF less was than ±1 mV. The ISE detection rate was 4–5 min/sample. ^2^ Time used for processing 12 soil samples.
